# Effect of food limitation and reproductive activity on fecal glucocorticoid metabolite levels in banded mongooses

**DOI:** 10.1186/s12898-020-00280-z

**Published:** 2020-02-18

**Authors:** Pete N. Laver, André Ganswindt, Stefanie B. Ganswindt, Kathleen A. Alexander

**Affiliations:** 1 Biodiversity and Development Institute, 4 Gunner’s Park, Grenville Avenue, Epping 1, Cape Town, 7460 South Africa; 2 Mammal Research Institute, Department of Zoology and Entomology, Faculty of Natural and Agricultural Sciences, University of Pretoria, Private Bag X20, Hatfield, 0028 South Africa; 3 Department of Anatomy and Physiology, Faculty of Veterinary Science, University of Pretoria, Private Bag X04, Onderstepoort, 0110 South Africa; 4 Centre for Veterinary Wildlife Studies, Faculty of Veterinary Science, University of Pretoria, Private Bag X04, Onderstepoort, 0110 South Africa; 5 Department of Fish and Wildlife Conservation, Virginia Tech, 100 Cheatham Hall, Blacksburg, VA 24061-0321 USA

**Keywords:** Homeostatic overload, Allostasis, *Mungos mungo*, *Mycobacterium mungi*

## Abstract

**Background:**

Glucocorticoids mediate responses to perceived stressors, thereby restoring homeostasis. However, prolonged glucocorticoid elevation may cause homeostatic overload. Using extensive field investigations of banded mongoose (*Mungos mungo*) groups in northern Botswana, we assessed the influence of reproduction, predation risk, and food limitation on apparent homeostatic overload (n=13 groups, 1542 samples from 268 animals). We experimentally manipulated reproduction and regulated food supply in captive mongooses, and compared their glucocorticoid responses to those obtained from free-living groups.

**Results:**

At the population level, variation in glucocorticoid levels in free-living mongooses was explained by food limitation: fecal organic matter, recent rainfall, and access to concentrated anthropogenic food resources. Soil macrofauna density and reproductive events explained less and predation risk very little variation in glucocorticoid levels. Reproduction and its associated challenges alone (under regulated feeding conditions) increased glucocorticoid levels 19-fold in a captive group. Among free-living groups, glucocorticoid elevation was seasonal (occurring in late dry season or early wet season when natural food resources were less available), but the timing of peak glucocorticoid production was moderated by access to anthropogenic resources (groups with fewer anthropogenic food sources had peaks earlier in dry seasons). Peak months represented 12- and 16-fold increases in glucocorticoids relative to nadir months with some animals exhibiting 100-fold increases. Relative to the captive group nadir, some free-living groups exhibited 60-fold increases in peak glucocorticoid levels with some animals exhibiting up to 800-fold increases. Most of these animals exhibited 1- to 10-fold increases relative to the captive animal peak.

**Conclusions:**

Banded mongooses exhibit seasonal chronic glucocorticoid elevation, associated primarily with food limitation and secondarily with reproduction. Magnitude and duration of this elevation suggests that this may be maladaptive for some animals, with possible fitness consequences. In late dry season, this population may face a convergence of stressors (food limitation, agonistic encounters at concentrated food resources, evictions, estrus, mate competition, parturition, and predation pressure on pups), which may induce homeostatic overload.

## Background

Glucocorticoids play a crucial but complex role in the evolutionary fitness of animals across many species. Glucocorticoid production exerts a critical influence on survival, reproductive success, and individual performance [1]. Glucocorticoids also play a key role in resource allocation trade-offs among life history traits [1], energetics, and immune function [2–4]. Glucocorticoid responses and their influences can be central to understanding species–environment interactions, providing information critical to understanding wildlife population needs in transforming landscapes. We face, however, major challenges in isolating these interactions because of the complexity surrounding (1) the complicated physiological roles glucocorticoids play in the body (action of glucocorticoids, below), (2) the diversity of interdependent factors eliciting glucocorticoid production, and (3) the consequences and costs of glucocorticoid production.

### Action of glucocorticoids

Glucocorticoid production is just one possible response to a stressor from among a suite of physiological responses in vertebrates. The prototypical endocrine part of a vertebrate stress response occurs in two “waves” and includes the following mediators (their tissue origin), and the general time-frame for production [4]:

Wave One, over the course of seconds:

1. Increased secretion of the catecholamines, epinephrine and norepinephrine (sympathetic nervous system); corticotropin-releasing hormone (hypothalamus); adrenocorticotropic hormone (pituitary); prolactin and growth hormone (pituitary); glucagon (pancreas).
2. Decreased secretion of gonadotropin-releasing hormone (hypothalamus); gonadotropins (pituitary).

These mediators elicit responses in their target tissues on the scale of seconds to minutes.

Wave Two, production over the course of minutes:

1. Increased secretion of glucocorticoids (adrenal cortex).
2. Decreased secretion of gonadal steroids (ovaries or testes).

Tissue effects of glucocorticoids occur after about an hour and may last for hours, while tissue effects of gonadal steroids are experienced on the scale of hours and days.

Glucocorticoids have four basic actions or roles including one as background or baseline role and three as part of the stress response [4]:

1. Permissive—glucocorticoid concentrations at baseline or background levels keep the animal “primed” to respond to challenges and facilitate Wave One (above) of the stress response when a stressor is encountered.
2. Suppressive—stressor-induced increases in glucocorticoid concentrations moderate the animal’s stress response (including Wave One) and prevent other mediators from “overshooting”.
3. Stimulating—stressor-induced increases in glucocorticoid concentrations enhance the effects of Wave One.
4. Preparative—stressor-induced increases in glucocorticoid concentrations affect the animal’s response to *future* stressors.

### Reactive scope model

In one of its primary roles, glucocorticoids mediate responses to physiological challenges [4], as modeled by the reactive scope model [5]. The reactive scope model [5] provides a conceptual framework for understanding stress responses, and it extends the concepts of allostasis (or “maintaining stability through change”) and allostatic load (the “cost” of an overactive or inefficient physiological response and “wear and tear” costs of normal stress responses plus facilitation of life history state changes) [6]. In the reactive scope model, physiological mediators of the stress response (e.g. glucocorticoids) are produced at concentrations that fall within four regions. Two of these regions constitute the “normal” reactive scope for that mediator, covering stress responses for predictable environmental change (e.g. seasons and daily rhythms) (predictive homeostasis) and unpredictable or threatening environmental change (up to some threshold) (reactive homeostasis). Above the upper threshold for reactive homeostasis, lies homeostatic overload. Below the lower threshold for predictive homeostasis, lies homeostatic failure. In both the homeostatic overload and homeostatic failure regions, the concentrations of the physiological mediator can have pathological effects.

The reactive scope model allows for “normal” seasonal and daily fluctuations in physiological mediators. When acute stressors elicit a physiological response below the homeostatic overload threshold, the animal has the capacity to respond appropriately. However, when acute stressors are repeated, or when stressors are prolonged, there are long-term costs of having an elevated concentration of the mediator. Depending on the size of the response, the frequency of repetition, or the duration of the response, the animal’s capacity or scope for an appropriate physiological response can become diminished—thus the reactive scope is reduced. This can result in either a short-term or long-term lowering of the homeostatic overload threshold, making it more likely that the same physiological stress response that previously fell within the reactive homeostasis region, now results in a pathology. In extreme cases of this process, the homeostatic overload threshold is lowered so far that it lies within the region of predictive homeostasis, and “normal” physiological mediator concentrations for maintaining homeostasis are now pathological. This leads to homeostatic failure.

### What elicits glucocorticoid production?

Many challenges elicit glucocorticoid production in wild animals, including food limitation [7], pregnancy [8], predation risk [9, 10], vigilance [11], sociality and group size [12–14], dominance hierarchies [15], anthropogenic disturbance [16], anthropogenic habitat change [17], direct anthropogenic food provisioning [18], climatic events [7], physical injury [19], and parasitism [20]. Food limitation is a common stressor leading to glucocorticoid responses, which then control energetic resources in animals [21], but the glucocorticoid-energetics relationship is itself complex. Where animals face progressive nutritional limitation, glucocorticoid production initially increases, facilitating gluconeogenesis (production of circulating glucose from non-carbohydrate substrates [e.g. proteins and lipids]), but later decreases as glucagon (pancreatic peptide hormone that increases circulating glucose by causing the liver to convert stored glycogen to glucose) production increases. Below critical body condition thresholds, glucocorticoid production again increases, facilitating protein catabolism [5, 7]. Increased glucocorticoid production in response to food limitation has been observed in several species in the field [12, 22–26] and through experimental manipulation [27–30].

In addition to food limitation, reproduction-glucocorticoid associations appear well-conserved across amphibians, reptiles, and birds, where chronic glucocorticoid elevations typically occur in a seasonal pattern (breeding season peak) [31]. However, seasonal glucocorticoid elevations in mammals show variability in timing relative to reproductive events [31]. Timing or seasonality of glucocorticoid production is important for understanding the ecological context of a stress response, and three (“seasonality-glucocorticoid”) hypotheses have been posited for why seasonal patterns in glucocorticoid production may exist. Firstly, the energy mobilization hypothesis predicts increased glucocorticoid production when energetic demands or deficits are greatest, because of metabolic effects of glucocorticoids (e.g. gluconeogenesis, above) [31]. Secondly, the behavior hypothesis predicts reduced glucocorticoid production during the breeding season, because of glucocorticoids mediating certain behaviors (e.g. movement out of a habitat during adverse conditions) [31]. Thirdly, the preparative hypothesis predicts increased glucocorticoid production during periods when the likelihood of stressors is increased (e.g. during periods of breeding or increased predation risk), because glucocorticoids mediate other stress response pathways (e.g. production of catecholamines, neurotransmitters, and cytokines) through permissive, stimulatory, suppressive, and preparatory effects [31] (see action of glucocorticoids, above). A reproductive peak in glucocorticoid production in mammals and the three seasonality-glucocorticoid hypotheses need further testing.

### What are the consequences of glucocorticoid production?

Beyond the complexity surrounding stressor–glucocorticoid relationships, the consequences of glucocorticoid elevation are not fully resolved either. For example, prolonged glucocorticoid elevations are presumed to be deleterious, but relationships between baseline glucocorticoid levels and fitness (e.g. reproduction and survival) are inconsistent [1, 32] and chronic stress responses in wild animals (days to weeks [33]) may be adaptive in some circumstances and maladaptive in others [33]. In maladaptive responses, chronically-elevated glucocorticoids may cause homeostatic overload [6]. To understand roles and consequences of glucocorticoid production at this level, we should view stress responses within the ecological contexts in which they occur as this may determine whether chronic stress is appropriate or maladaptive [33, 34]. Critical elements include timing, duration, and magnitude of the stress response, individual traits (e.g. sex, age, life history stage), and putative cause (stressor or challenge). Thus, resolving the causes, physiological roles, and long-term consequences of glucocorticoid production is challenging, but the reactive scope model [5] provides the necessary theoretical framework for engaging this complexity.

### Identifying stressors and scale of associated glucocorticoid responses

Practical considerations compound the theoretical complexities indicated above. Identifying chronic stress may pose challenges [35], as does identifying stress responses that have ecologically-relevant effect sizes. Within ecological systems, multiple challenges interact, complicating the identification of stressors. For example, energy deficit and associated glucocorticoid production may be assigned to food limitation but actually arise from reproductive activity or agonistic interactions, both of which are energetically costly for mammals [31, 36, 37]. Further, chronic stress responses are integrated over time periods that permit multiple simultaneous or consecutive stressors. Thus, experimental studies controlling multiple causes enable robust inferences about particular stressors, but may overlook complex, potentially synergistic challenges facing free-living animals.

Our primary aim was to resolve the relationships of multiple putative stressors with glucocorticoid production, while these stressors acted in concert, in a free-living population of mammals. We combined a long-term observational study and experimental approaches to evaluate concentrations of fecal glucocorticoid metabolites (fGCMs) [38] in relation to potentially synergistic stressors within free-living banded mongooses (*Mungos mungo*) in northern Botswana. Using fGCMs integrates stress responses over longer periods (approximately 24 h, the gastrointestinal transit time [38]) than serum measurements, and allows for non-invasive assessment of many animals at broad spatio-temporal scales, thereby permitting modeling of chronic stress responses and the complex milieu of stressors challenging free-living wild animals. We assessed glucocorticoid levels of most members of several mongoose groups (n = 13), repeatedly over several years (2008–2011), and hence make inferences at group and population levels. These free-living social groups occurred along a gradient of association with humans (synanthropy) [39], which we use for context in understanding our results.

Our ancillary experimental approach, suppressing reproductive activity in and providing constant daily food supply to a group of captive banded mongoose, allowed us to partial out effects of different stressors and provided context (relative effect sizes and timing of glucocorticoid production) for understanding the revealed glucocorticoid patterns. Comparisons between the free-living and captive mongoose fGCMs should be interpreted with caution as glucocorticoid responses to stressors may differ among captive and free-living individuals [40–42].

We asked the following questions: (1) Do banded mongooses experience chronic elevations of glucocorticoids? (2) What is the context for glucocorticoid elevations with regard to their timing, duration, and effect size? Lastly, (3) Which ecological covariates best explain variability in determined glucocorticoid levels among groups of free-living mongooses in this system? Based on literature concerning stressors in banded mongoose and other taxa, we investigated the influence of food limitation, reproduction, and predation risk on banded mongoose fGCMs, each with covariates specific to our study system (Table 1). This selection of candidate variables was justified *a priori*:

1. Food limitation: Food limitation explains variability in glucocorticoid levels in several vertebrate taxa [7, 12, 22–30]; our study site is dystrophic with dramatic seasonality in rainfall and primary production; mongoose groups at this site have variable access to anthropogenic food and modify space use in response to anthropogenic food availability (groups with access to lodge trash sites cluster around these sites during the dry season when natural food resources are less available and lodge occupancy [and hence, food waste production] is higher; groups without access to anthropogenic food resources expand their space use during the dry season) [39]. Alloparental investment (provisioning pups) by banded mongoose carers is associated with energetic losses and a corresponding increase in fGCMs [43]. Supplementary feeding in banded mongoose carers or escorts (but not non-escorts) lowered fGCM concentrations [43].
2. Reproduction: The reproduction-glucocorticoid relationship requires further testing in mammals [31], and is complicated by reproduction encompassing estrus, mate guarding, mating, pregnancy, parturition, lactation and parental care. In banded mongooses, evictions from groups and associated agonistic encounters occur during estrus [44, 45] or more generally during breeding attempts [46]. Banded mongooses also share parental care costs, some of which may be energetic [43]: escorts provision pups nearest to them [47]; males share guarding of altricial young [48]; females allosuckle [49]. Alloparental care is associated with increased fGCMs in banded mongoose, although this may be driven by energetics [43]. Lower ranked female banded mongooses have higher fGCMs later in pregnancy, although this may also be driven by energetic differences during gestation, specifically, exclusion of subordinates from food resources by higher ranked females [50].
3. Predation risk: Predation risk affects group behavior, whereby banded mongooses respond to predator threats and rival groups through group mobbing responses [51]. Although banded mongooses breed communally because of benefits of rearing young cooperatively and lack of inbreeding costs [52], other herpestids such as meerkats (*Suricata suricatta*) benefit from group-mediated anti-predator behavior [53]. It is thus reasonable to assume that predation risk may elicit glucocorticoid production in banded mongooses.

We predicted negative association between fGCMs and four proxies for food limitation (Table 1). We also predicted positive association between fGCMs and reproduction, and negative association between fGCMs and covariates reducing individual predation risk (Table 1). We used our results to explore our current theoretical understanding of the glucocorticoid-mediated stress response in banded mongooses, including the ecological relevance of the measured effect sizes, whether the observed stress responses were appropriate or maladaptive, and which seasonality-glucocorticoid hypothesis our data provide support for. We also discuss possible fitness consequences for chronic glucocorticoid elevation in this population.

**Table 1:** Predicted association, and justification of candidate variables used in modeling baseline fecal glucocorticoid (GC) metabolite concentrations

Putative fixed effect and justification, mechanism, or example	Predicted association with GCs
A. Food limitation	
1. General	
(a) GCs increase in response to natural food limitation	
(i) GCs increase in the dry season and are negatively correlated with rainfall in African elephant (*Loxodonta africana*) [12]	
(ii) GCs were higher in a food-limited group versus a food-abundant group in black-legged kittiwakes (*Rissa tridactyla*) [25]	
(b) GCs increases in response to experimental food limitation	
(i) GCs increase under food limitation in black-legged kittiwakes (*Rissa tridactyla*) [27]	
(ii) Food limitation during development increases GCs in western scrub-jays (*Aphelocoma californica*) [28]	
(iii) Food limitation and unpredictability increase GCs in mountain chickadees (*Poecile gambeli*) [29]	
(iv) Food limitation increases GCs in barn swallows (*Hirundo rustica*) [30]	
2. Access to anthropogenic food resources	Negative
(a) GCs decrease during anthropogenic food provisioning in Sykes’ monkeys (*Cercopithecus mitis albogularis*) [18]	
(b) Refuse-feeding banded mongooses exhibit better physical condition than non-refuse-feeders [54]	
(c) Banded mongoose area use is concentrated around refuse sites [39, 55]	
(i) GCs increase with increased foraging travel time in Mexican howlers (*Alouatta palliata mexicana*) [23]	
(ii) GCs increase under high food search demand effort in squirrel monkeys (*Saimiri sciureus*) [22]	
(d) Banded mongoose escorts lose body mass while provisioning pups and exhibit increased fGCMs, but fGCMs are reduced in these animals if fed supplementally [43]	
3. Fecal organic matter	Negative
(a) Indicator of organic matter intake in cattle (*Bos taurus*) and goats (*Capra aegagrus*) [56]	
(b) Complementary measures, fecal ash and ingested soil, also indicate food limitation	
(i) Domestic sheep (*Ovis aries*) increase soil ingestion as forage [57] and food supplementation [58, 59] decrease and stocking rates increase [60]	
(ii) Aardwolves (*Proteles cristata*) have more fecal sand when termites are scarce [61]	
(iii) Tamanduas (*Tamandua tetradactyla*) ingest more substrate during behavioral or dietary deficits [62]	
(iv) Three-banded armadillos (*Tolypeutes tricinctus*) ingested more soil in dry seasons [63]	
(v) Giant anteater (*Myrmecophaga tridactyla*) fecal nutrition markers were inversely related to fecal ash [64]	
4. Recent rainfall	Negative
(a) Millipedes and (at times) termite alates dominate banded mongoose diet [65]	
(b) Rainfall affects banded mongoose prey availability: soil macroinvertebrates [66]; millipedes [67]; termite alates [68]	
(i) Residual effect of rain on millipede availability may last up to 8 days [67]	
5. Soil macrofauna density	Negative
(a) Soil macrofauna densities at our study site vary by habitat type [66]	
B. Reproduction	
1. Breeding status	Positive
(a) GCs increase in female meerkats (*Suricata suricatta*) as pregnancy progresses [8]	
(b) GCs increase in mate-guarding male long-tailed macaques (*Macaca fascicularis*) [69]	
(c) Mating, parturition, associated agonistic encounters, and pup depredations increase GCs in captive banded mongooses [38]	
(d) Alloparental care (pup provisioning) in banded mongoose is associated with increased fGCMs (although this may be driven by energetic losses) [43]	
(e) Subordinate female banded mongoose have higher fGCM concentrations in late pregnancy than higher ranked females (although this may be driven by exclusion from food resources and resulting energetic losses) [50]	
C. Predation risk	
1. Group size	Negative
(a) GCs increase under higher predation risk in European rabbits (*Oryctolagus cuniculus*) [10]	
(i) Larger groups should lower per capita predation risk—dilution effect [70–73]	
(ii) Larger groups should lower per capita vigilance—detection effect [74, 75]	
(iii) Larger group sizes do exhibit lower per capita vigilance in banded mongooses [76]	
(iv) GCs are positively associated with vigilance in meerkats [11]	
2. Canopy cover	Negative
(a) Aerial predators are putatively important, if not predominant natural predators of banded mongooses e.g. martial eagles (*Polemaetus bellicosus*) [77]	
(i) Hunting success for large raptors is diminished in areas of higher canopy cover e.g. Bonelli’s eagle (*Aquila fasciata*) [78, 79]	

Candidate models modeled fecal glucocorticoid metabolite concentrations in banded mongooses (*Mungos mungo*), northeastern Botswana (2008–2011)

## Results

### Models of variability in fecal glucocorticoid metabolite levels

**Fig. 1:**
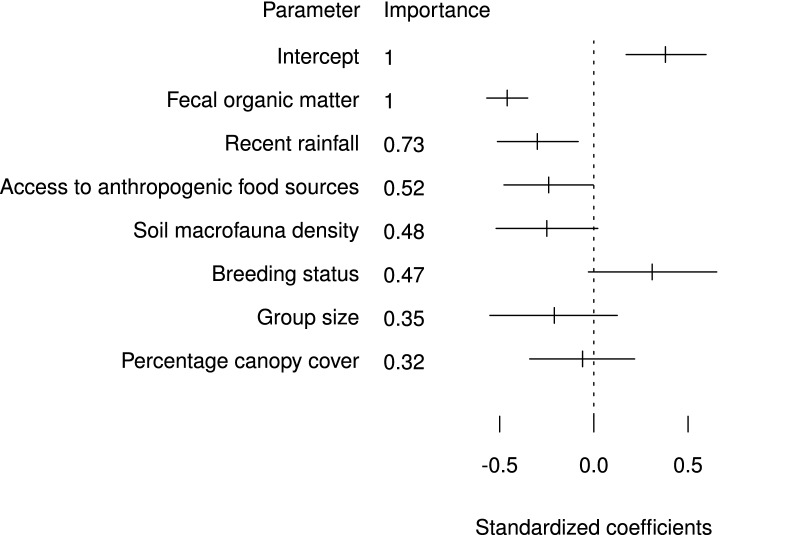
Effect sizes of covariates describing glucocorticoid metabolite concentrations in free-living banded mongooses. Model averaging, parameter estimation (effect sizes), 85% confidence intervals [80], and relative importance [sum of Akaike weights ($\Sigma w_i$)] for standardized ecological covariates describing fecal glucocorticoid metabolite (fGCM) variability in banded mongooses (*Mungos mungo*), northeastern Botswana (2008–2011)

**Fig. 2:**
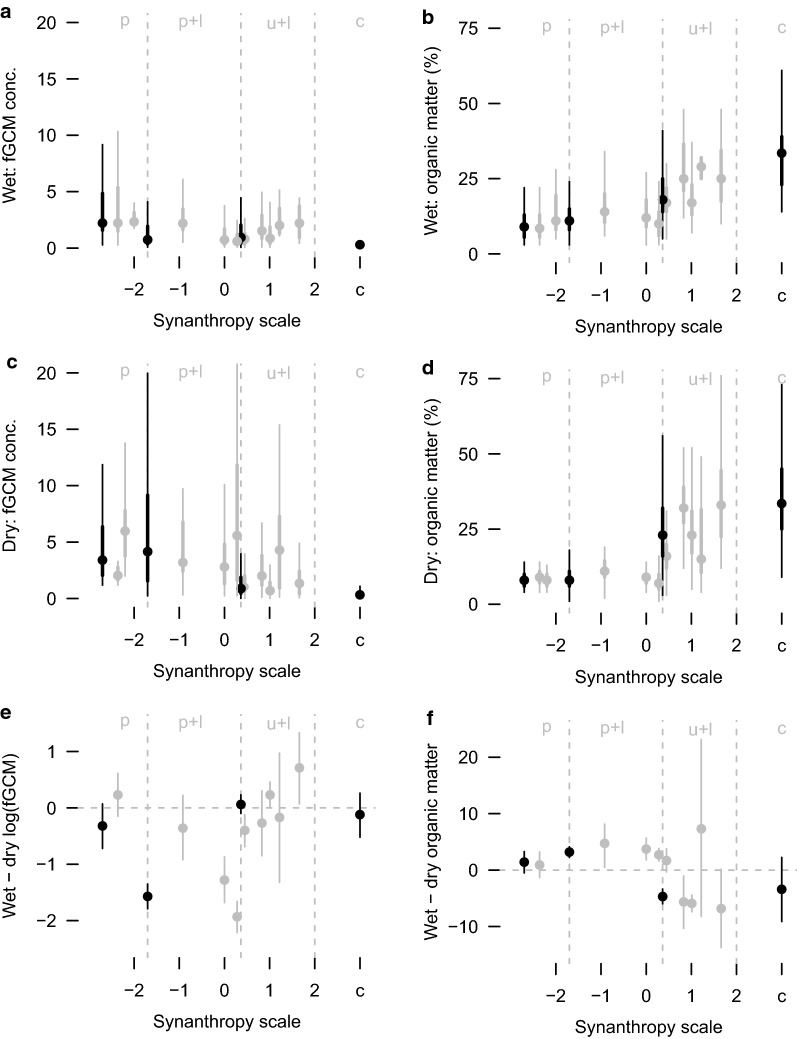
Effects of food limitation, season, and access to concentrated anthropogenic food resources on glucocorticoid metabolites. Quartile plots (thick lines: interquartile range; thin lines: range; point: median) (**a**–**d**) and Bayesian estimation (means, points, with highest posterior density intervals, HPDI, lines) (**e**,** f**) of wet season (**a**,** b**), dry season (**c**,** d**), and seasonal differences (**e**, ** f**) in fecal glucocorticoid metabolite (fGCM) concentrations (**a**,** c**,** e**) and percentage fecal organic matter (**b**,** d**, ** f**) in banded mongoose (*Mungos mungo*) groups, northeastern Botswana (2008–2011). Values are shown for groups (gray dots and lines) along a synanthropic scale (increasing access to anthropogenic resources from left to right, ending with a captive group). Broad categories (black dots and lines) are indicated along this scale for “park” (p), “park + lodge” (p + l), and “urban + lodge” (u + l). Results for the Bayesian estimation (**e**, ** f**) are provided in Tables 3 and 4

Proportion of fecal organic matter, rainfall, and group access to concentrated anthropogenic food resources explained variability in fecal glucocorticoid metabolite levels (Table 2, Fig. 1), with fecal organic matter being most important (summed Akaike weight, $\Sigma w_i = 1$) (Fig. 1). These effects are interpreted graphically in Fig. 2 and in Tables 3 and 4. We selected fecal organic matter in all, and rainfall in all but one, of our best candidate models ($\Delta$AIC_*c*_ < 2, Table 2). These variables also had the largest standardized effect sizes after model averaging (Fig. 1). Other covariates were of low importance, had high variability in parameter estimates, or had small standardized effect sizes (Fig. 1). Our global model explained 55% of variation in fGCMs, with $\Omega _0^2 = 0.55$. Variance inflation factors for all covariates were below 2.

**Table 2:** Information-theoretic model selection results for all free-living banded mongoose groups

Model: *ln*(fGCM) $\sim$	log*L*	*K*	AIC$_c$	$\Delta$	$w_i$
org + rain + anth	− 2176.5	7	4367.1	0.0	0.06
org + rain	− 2177.7	6	4367.4	0.4	0.05
org + rain + anth + breed	− 2175.7	8	4367.6	0.5	0.04
org + rain + breed	− 2176.8	7	4367.7	0.6	0.04
org + rain + anth + breed + macro	− 2174.8	9	4367.8	0.7	0.04
org + rain + anth + macro	− 2176.0	8	4368.1	1.0	0.03
org + rain + breed + macro	− 2176.1	8	4368.3	1.2	0.03
org + rain + anth + size	− 2176.2	8	4368.4	1.3	0.03
org + anth + macro	− 2177.2	7	4368.5	1.4	0.03
org + rain + cc	− 2177.2	7	4368.5	1.4	0.03
org + rain + anth + breed + size + macro	− 2174.2	10	4368.5	1.4	0.03
org + rain + anth + breed + size	− 2175.3	9	4368.7	1.6	0.02
org + rain + size	− 2177.3	7	4368.8	1.7	0.02
org + rain + macro	− 2177.4	7	4368.8	1.7	0.02
org + rain + breed + size	− 2176.4	8	4368.8	1.7	0.02
org + rain + breed + cc	− 2176.4	8	4368.9	1.8	0.02
org + rain + breed + size + macro	− 2175.4	9	4369.0	1.9	0.02

Fecal glucocorticoid metabolites (fGCMs) (n = 1542 feces), in free-living banded mongooses (*Mungos mungo*) in northeastern Botswana (2008–2011), were modeled by group identity (1|group) and sampling event (1|event) as random effects in all models (omitted from table), and fixed effects: percentage fecal organic matter (org); soil macrofauna density (macro); recent rainfall (rain); access to concentrated anthropogenic food sources (anth); breeding status (breed); group size (size); and percent canopy cover (cc). We analyzed all subsets for model averaging, but present only the best models ($\Delta \hbox {AIC}_c < 2$) here

**Table 3:** Seasonal differences in fecal glucocorticoid metabolite levels for banded mongoose groups

Category group	PC1	n		$\beta$	HPDI	Probability (%)	
		Wet	Dry			$\beta <0$	$\beta >0$
Captive							
1		32	66	− 0.12	− 0.52 to 0.26	72.7	27.3
Urban + lodge							
2	1.66	19	59	0.71	0.07–1.33	1.4	98.6
3	1.22	6	15	− 0.17	− 1.32 to 0.97	62.5	37.5
4	1.01	173	411	0.23	0.02–0.46	1.9	98.1
5	0.83	31	73	− 0.27	− 0.85 to 0.30	82.7	17.3
6	0.45	73	118	− 0.40	− 0.69 to − 0.12	99.7	0.3
		302	676	0.06	− 0.10 to 0.23	23.6	76.4
Park + lodge							
7	0.28	107	171	− 1.93	− 2.21 to − 1.66	100.0	0.0
8	0.00	52	65	− 1.28	− 1.68 to − 0.87	100.0	0.0
9	− 0.92	24	19	− 0.36	− 0.92 to 0.22	89.3	10.7
		183	255	− 1.57	− 1.79 to − 1.35	100.0	0.0
Park							
10	− 2.36	44	15	0.23	− 0.15 to 0.61	11.5	88.5
		47	34	− 0.32	− 0.72 to 0.07	94.7	5.3

Bayesian estimation ($\beta$, highest posterior density interval [HPDI], and Bayesian probabilities) for the seasonal difference (wet season minus dry season) between log-transformed fecal glucocorticoid metabolite concentrations within a group, arranged along a decreasing synanthropy scale (principal component [PC1], association with humans: see [39]). Combined analyses for broad categories are below the horizontal lines and may include groups not already listed (e.g. for groups that had data for only one season)

**Fig. 3:**
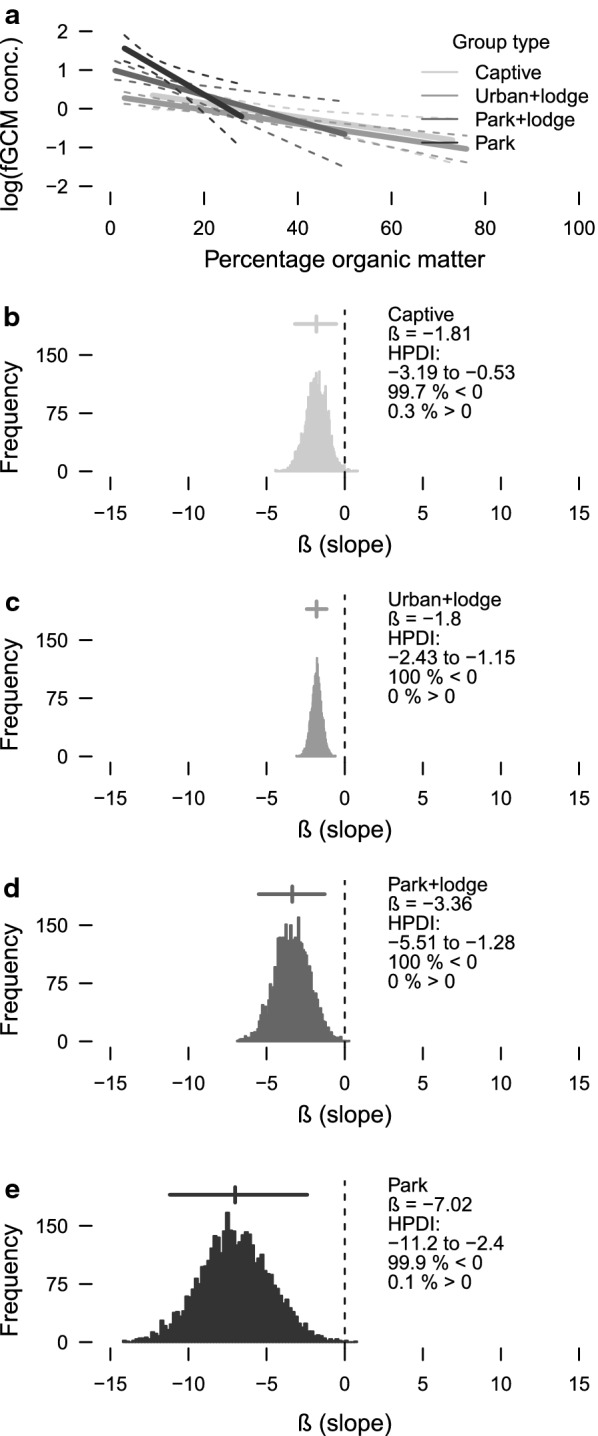
Associations between fecal glucocorticoid metabolite (fGCM) concentrations and fecal organic matter content in banded mongooses (*Mungos mungo*), northeastern Botswana (2008–2011). Effect sizes (model slopes, solid lines; with credible intervals, dashed lines) were larger for group categories with progressively less access to anthropogenic food resources (captive to “urban + lodge” to “park + lodge” to park) (**a**). Bayesian posterior distributions, mean effect size (slope, $\beta$), the highest posterior density interval (HPDI, horizontal line above each distribution), and the probability of the slope differing from zero are indicated for each group type (**b**–**e**)

Fecal organic matter provided a suitable proxy for food limitation. A mixed-effects model with this covariate performed better in explaining fGCM variability than did a random-effects model controlling only for repeated sampling (the different individuals within a group) on a given day. However, this model performance occurred for a free-living (“urban + lodge”) group but not for the captive group (fed a regular diet) (i.e., fecal organic matter explained fGCM variability better in free-living animals than captive animals). Analyses for both groups had similar sample sizes and sample collection schedules and the groups were in the same part of the study area. For the free-living group, we selected the mixed model outright ($w_i = 1$) (Table 5), which explained 54% of variation in fGCMs ($\Omega _0^2 = 0.54$), suggesting that other covariates were missing. For the captive group’s non-reproductive period, the mixed and random models both had some Akaike weight (Table 5). The mixed model had a relatively low evidence ratio ($w_i/w_j$) of 4.9 (Table 5) and explained 77% of variation in the captive group’s fGCMs ($\Omega _0^2 = 0.77$), while the random model was within 4 $\Delta$AIC$_c$ units of the mixed model and explained 76% of fGCM variation ($\Omega _0^2 = 0.76$). For the captive group the addition of fecal organic matter to the random model only marginally improved log-likelihood. The relative performance of fecal organic matter in this modeling framework contrasted starkly between one group subject to food limitation and another group fed a regular diet.

**Table 4:** Seasonal differences in fecal organic matter for banded mongoose groups

Category group	PC1	n		Mean (%)		$\beta$ (%)	HPDI (%)	Probability (%)	
		Wet	Dry	Wet	Dry			$\beta <0$	$\beta >0$
Captive									
1		32	66	32	36	− 3.41	− 9.12 to 2.24	88.2	11.8
Urban + lodge									
2	1.66	19	59	27	34	− 6.83	− 13.75 to 0.13	97.3	2.7
3	1.22	6	15	29	21	7.32	− 8.21 to 23.15	15.4	84.6
4	1.01	173	411	18	24	− 5.91	− 7.39 to − 4.44	100.0	0.0
5	0.83	31	73	28	33	− 5.65	− 10.33 to − 1.06	99.1	0.9
6	0.45	73	118	18	17	1.68	− 0.33 to 3.75	5.2	94.8
		302	676	19	24	− 4.69	− 5.98 to − 3.36	100.0	0.0
Park + lodge									
7	0.28	107	171	10	7	2.71	1.61–3.84	0.0	100.0
8	0.00	52	65	12	9	3.73	1.81–5.67	0.0	100.0
9	− 0.92	24	19	15	11	4.73	0.48–8.14	1.3	98.7
		183	255	11	8	3.19	2.23–4.12	0.0	100.0
Park									
10	− 2.36	44	15	10	9	0.90	− 1.30 to 3.20	21.2	78.8
		47	34	10	8	1.41	− 0.50 to 3.27	7.0	93.0

Bayesian estimation ($\beta$, highest posterior density interval [HPDI], and Bayesian probabilities) for the seasonal difference (wet season minus dry season) between fecal organic matter content within a group, arranged along a decreasing synanthropy scale (principal component [PC1], association with humans: see [39]). Combined analyses for broad categories are below the horizontal lines and may include groups not already listed (e.g. for groups that had data for only one season)

**Fig. 4:**
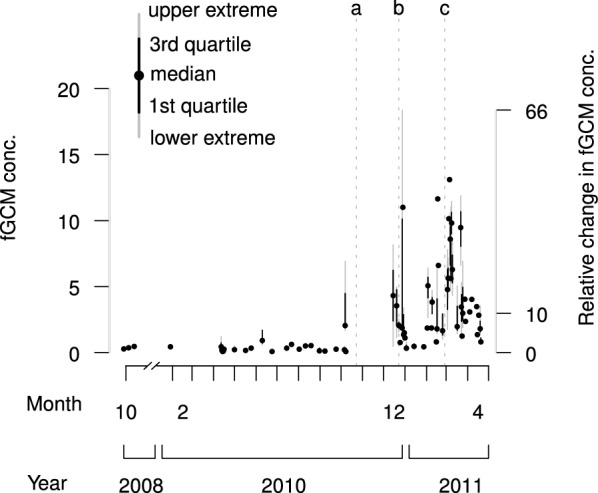
Effect of reproduction on fecal glucocorticoid metabolite (fGCM) concentrations in the absence of food limitation. Longitudinal profile of median fGCMs ($\upmu$g/g org. content) for each sampling day, in captive banded mongooses (*Mungos mungo*) (one female, three males), northeastern Botswana (2008–2011). Before May 2010 we suppressed estrus and observed no reproductive behavior until mating (October 2010, dotted line a). During their reproductive period, the female bore two litters (dotted lines b and c) and the group suffered predation and group invasions. Median fGCMs for the non-reproductive baseline and reproductive periods differed 10-fold (secondary *y*-axis: change relative to non-reproductive baseline). Portions of this dataset are summarized elsewhere [38]

**Fig. 5:**
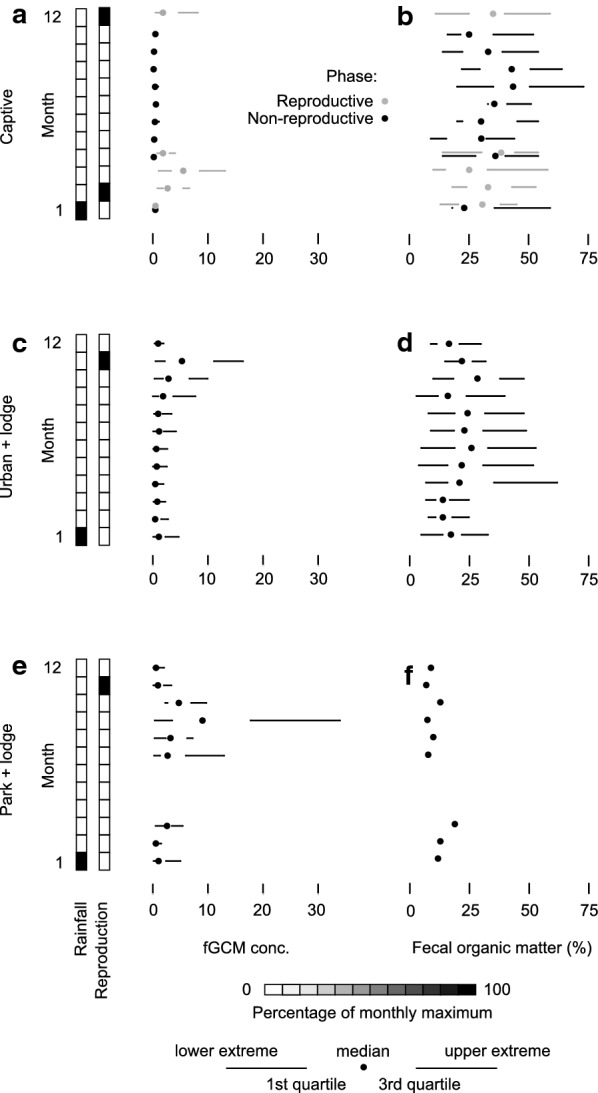
Monthly effects of food limitation, anthropogenic food, reproduction, and rainfall on fecal glucocorticoid metabolites (fGCMs). Quartile plots [81] of fGCMs ($\upmu$g/g org. content) (**a**,** c**,** e**), and percentage fecal organic matter (**b**,** d**,** f**), by month for categories of banded mongoose (*Mungos mungo*) groups, northeastern Botswana (2008–2011). We categorized mongoose groups by decreasing exposure to anthropogenic resources, from “captive” (**a**,** b**) to “urban + lodge” (**c**, ** d**) to “park + lodge” (** e**, ** f**). We depict monthly rainfall with shading relative to the maximum median monthly rainfall (January: 184 mm) and monthly reproductive events (group estrus or parturition) relative to the monthly maximum number of reproductive events

A simple linear Bayesian regression (Fig. 3) of the association between fecal organic matter content and log-transformed fGCM concentration for the different categories of mongoose groups bore out the mixed-effects model results (above). The association between fecal organic matter and log-transformed fGCM concentration was negative for all categories of mongoose groups (Fig. 3a) (i.e. animals with more fecal organic matter had lower fGCM concentration). The association (effect size or slope) was weak for the captive group (Fig. 3b) and the “urban + lodge” groups (Fig. 3c). The magnitude of the effect for the “park + lodge” groups (Fig. 3d) was nearly double (0.8-fold larger) that of the captive group. The magnitude of the effect for the “park” groups (Fig. 3e) was 2.9-fold larger than the captive group and double that of the “park + lodge” groups (onefold larger). Thus, the putative effect of fecal organic matter on fGCM concentration became stronger in groups with less access to anthropogenic food resources. There was higher certainty in the “urban + lodge” result (narrower highest density posterior interval) (Fig. 3c) than in the captive group result (Fig. 3b), which may explain why the mixed model (above) performed so much better than the random model for the one free-living group compared to the captive group (Table 5). Relative to untransformed data, these effect sizes represented decreases in fGCM concentrations of 0.06, 0.07, 0.31, and 0.71 $\upmu$g/g org. content for 1% increases in fecal organic matter content for the captive group, “urban + lodge” groups, “park + lodge” groups, and park groups, respectively.

**Table 5:** Information-theoretic model selection results for one free-living and one captive group of banded mongooses

Group	Model: *ln*(fGCM) $\sim$	log*L*	*K*	AIC$_c$	$\Delta$	$w_i$
Free-living	org + (1|event)	− 783.8	4	1575.7	0.0	1.00
	(1|event)	− 797.5	3	1600.9	25.3	0.00
Captive	org + (1|event)	− 93.1	4	194.7	0.0	0.83
	(1|event)	− 95.8	3	198.0	3.2	0.17

Fecal glucocorticoid metabolites (fGCMs), in a free-living group (n = 584 feces) and a captive group (n = 86 feces) of banded mongooses (*Mungos mungo*), northeastern Botswana (2008–2011), modeled by sampling event (1|event, random effect) and percentage fecal organic matter (org, fixed effect)

### Timing, magnitude, duration, and seasonality of fecal glucocorticoid metabolite elevations

While fed a regular diet, and with the one captive female reproductively-suppressed (using a contraceptive), no overt seasonal fGCM response could be detected among the four captive animals (Fig. 4). They had low fGCM concentrations with low variability (n = 98 from four animals, median = 0.28 $\upmu$g/g org. content, inter-quartile range = 0.37 $\upmu$g/g org. content) [38]. During their reproductive period, they had 10-fold higher fGCM concentrations with higher variability (n = 104 from four animals, median = 2.98 $\upmu$g/g org. content, inter-quartile range = 4.37 $\upmu$g/g org. content) [38]. Peak fecal glucocorticoid responses occurred shortly after parturition and coincided with behavioral estrus, mating, pup depredation by an African rock python (*Python sebae natalensis*), group invasion by foreign males, and loss of three of four pups from a second litter (Fig. 4). Glucocorticoid responses to these events were described for individual animals elsewhere [38]. fGCMs also increased leading up to parturition: after loss of the first litter and removal of foreign males, fGCMs increased fivefold from January 2011 (n = 14, median = 0.45 $\upmu$g/g org. content, inter-quartile range = 0.28 $\upmu$g/g org. content) to February 2011 (n = 21, median = 2.64 $\upmu$g/g org. content, inter-quartile range = 3.68 $\upmu$g/g org. content), unrelated to discernible external challenges (agonistic encounters or predation).

**Fig. 6:**
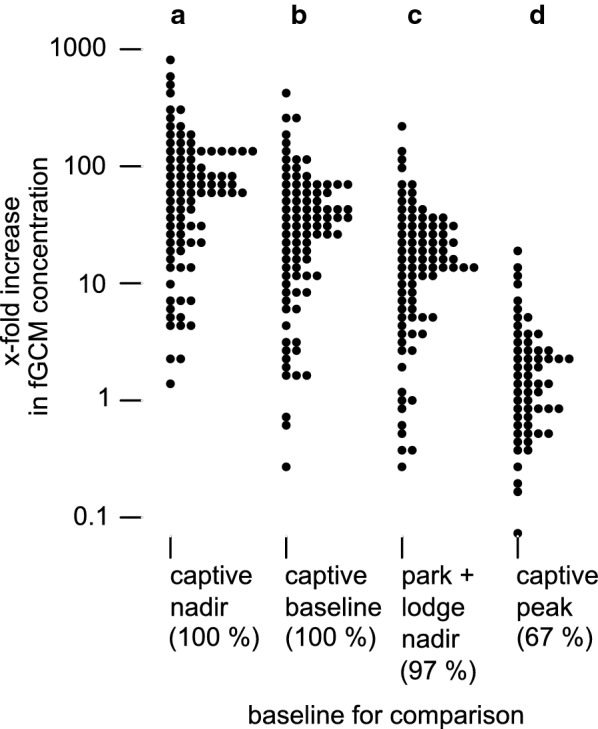
Relative increase in peak month fecal glucocorticoid metabolite (fGCM) concentrations. Dot histograms of fGCM concentrations during the peak month in free-living banded mongooses (*Mungos mungo*) from categories of groups living at single lodges in Chobe National Park (“park + lodge”) relative to the median fGCM concentrations in (a) the nadir month for a captive group, (b) the long-term non-reproductive baseline for a captive group, (c) the nadir month for the same category of “park + lodge” groups, and (d) the peak month for a captive group. The majority of fecal samples (percentage listed) had higher fGCM concentrations than the comparison value. Note the logarithmic y-axis

**Fig. 7:**
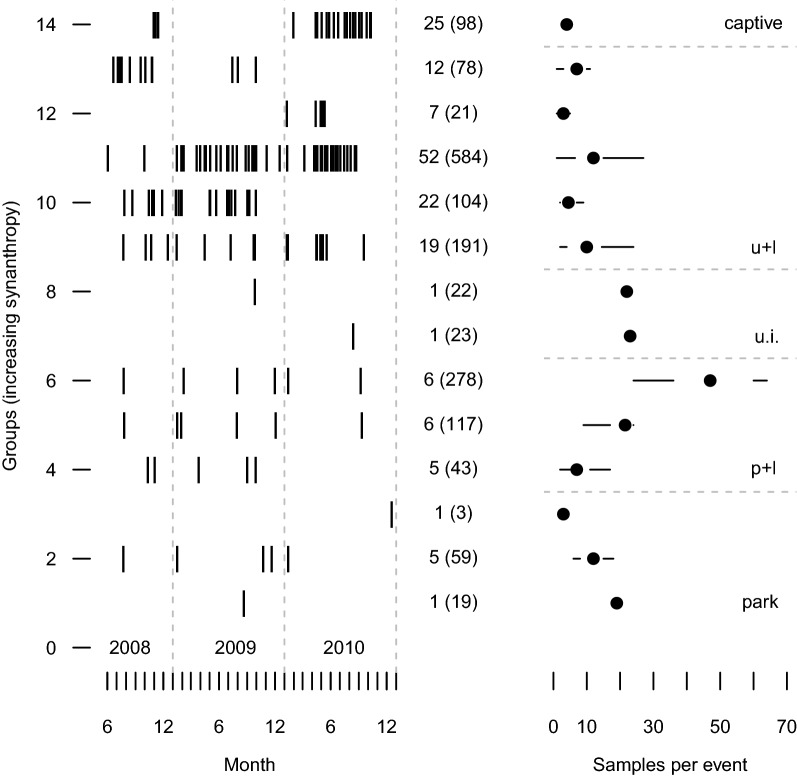
Schedule and sample sizes for fecal sampling 2008–2010. Sampling dates, number of sampling events (with total sample tally) and quartile plots of sample sizes per event for 14 groups of banded mongooses (*Mungos mungo*) in northern Botswana, 2008–2010. Groups are indicated in increasing order of synanthropy (association with humans) along with broad categories: “park”, “park + lodge” (p + l), “urban interface” (u.i.), “urban + lodge” (u + l), and “captive”

With a regular food supply, reproduction and its associated physiological challenges appeared to drive fGCM concentrations in captive animals (Figs. 4, 5a). Reproductive activity and fGCM concentrations followed a seasonal pattern (Fig. 5a). However, in free-living groups, fGCM concentrations increased in late dry season without following the seasonal reproduction-related pattern seen in the captive group (i.e., continued wet season fGCM elevation for subsequent reproductive events throughout the breeding season) (Fig. 5c, e). Peak fGCM responses in “urban + lodge” groups occurred in November, the approximate wet season start and time of first parturition (Fig. 5c). For “park + lodge” groups, peak fGCM responses occurred in September at the approximate time of first estrus, eviction and dispersal (Fig. 5e). Although the timing of peak and nadir months for fGCMs in “park + lodge” and “urban + lodge” free-living groups coincided approximately (peak: September and November; nadir: February), this timing was reversed in the captive group (peak: March; nadir: September) (Table 6).

**Table 6:** Relative magnitude (in *x*-fold change) of stress responses in categories of banded mongoose groups, northeastern Botswana (2008–2011)

Group	Month	fGCM	Captive			Urban + lodge		Park + lodge
			Nadir	Baseline	Peak	Nadir	Peak	Nadir
			0.14	0.28	5.49	0.41	5.28	0.55
Captive	Nadir (9)	0.14						
	Baseline	0.28	+ 1					
	Peak (3)	5.49	+ 40	+ 19				
Urban + lodge	Nadir (2)	0.41	+ 2	+ 0.5	− 12			
	Peak (11)	5.28	+ 38	+ 18	− 0.04	+ 12		
Park + lodge	Nadir (2)	0.55	+ 3	+ 0.9	− 9	+ 0.3	− 9	
	Peak (9)	9.01	+ 66	+ 31	+ 0.6	+ 21	+ 0.7	+ 16

Stress responses in nadir, baseline, and peak months (with month number) as measured by fecal glucocorticoid metabolites (fGCMs, monthly median, $\upmu \hbox {g}/\hbox {g}$ org. content), in free-living and captive banded mongooses (*Mungos mungo*). We include the long-term non-reproductive baseline for the captive group. Reading across rows, values within the matrix represent the *x*-fold change in fGCMs from the first column relative to the corresponding fGCM value on the top row (e.g. the captive group’s baseline was double [onefold increase] its nadir). *x*-fold values $> 1$ were rounded to the nearest whole number

We had frequent sampling over the entire study period for one of the “park + lodge” groups to obtain a detailed longitudinal profile. This group had access to several lodges and their refuse sites and had relatively low dry season fGCM concentrations compared to other groups. Nonetheless, during the dry season, the group median fGCM concentration was approximately two- to six-fold higher than the nadir month’s value for the “park + lodge” category. This elevation was evident for approximately 2 months at the end of the dry season.

The effect sizes (relative differences in fGCM concentrations) among the different categories of mongoose groups were large, even when using just the median value for a group for either the nadir, baseline, or peak month in their fGCM profiles (Fig. 5, Table 6). fGCM concentrations in peak months within a group were 12- to 40-fold higher than nadir months (Table 6). Free-living mongooses during their nadir months exhibited two- to threefold higher fGCM concentrations than captive animals during the captive nadir, and 0.5- to 0.9-fold higher fGCM concentrations than the captive animals during the captive long-term non-reproductive baseline (Table 6). Free-living mongooses during their peak months either had similar fGCM concentrations (0.04-fold decrease) or had 0.6-fold higher fGCM concentrations relative to the captive peak (Table 6).

The median fGCM concentration (as described above) represents just the middle value in a group, and for half the members of these groups, the effect sizes are even larger. For example, the full distribution of fGCM concentrations in the “park + lodge” category during its peak month suggests that many animals exhibited extreme glucocorticoid elevation relative to various baseline measures, with some animals exhibiting up to 800-fold increases relative to the captive group’s nadir (Fig. 6a–c). Most members of the group during the peak month had fGCM concentrations that were 10- to 100-fold higher than the captive baseline (Fig. 6a–c). Even relative to the captive group’s peak month, four “park + lodge” animals exhibited more than 10-fold relative increases in fGCM concentrations and most of the group had fGCM concentrations that were at least double (onefold increase) those of the captive peak (Fig. 6d).

### Seasonality in fecal organic matter

For mongoose groups with access to anthropogenic resources, monthly fecal organic matter levels exhibited high variability but lacked the clear seasonal pattern (Figs. 2b, 5b, d, f) exhibited in fGCM concentrations (Figs. 2a, 5a, c, e). However, most groups had wet and dry season fecal organic matter contents that differed (Table 4). The effect sizes (differences between seasons) were small and the direction of effect appeared to depend on access to anthropogenic food resources (Table 4). The captive group appeared to have higher fecal organic matter in the dry season than the wet season, but there was high variability in their fecal organic matter between seasons and we had low certainty in this outcome (HPDI: − 9.1 to 2.2%, $p(\beta < 0)=88 \%$, Table 4). The “urban + lodge” category had higher dry than wet season fecal organic matter (HPDI: − 6 to − 3.4%, $p(\beta < 0)=100 \%$, Table 4), but the “park + lodge” category had lower dry than wet season fecal organic matter (HPDI: 2.2 to 4.1%, $p(\beta > 0)=100 \%$, Table 4), as did the “park” category (HPDI: − 0.5 to 3.3%, $p(\beta > 0)=93 \%$, Table 4).

### Reproductive events

We observed first emergence of neonates from dens at the beginning of the breeding season on 15 occasions for ten groups (five groups, 2 years each; five groups, 1 year each). Backdating these events to putative conception or estrus dates, we estimated that the first matings of breeding seasons occurred in August five times and in September ten times. We also observed first matings (twice) and mate-guarding (once) at the beginning of the season. All three events occurred in September. Within a group, pup emergence dates differed by 1 to 15 days (median, 6 days) among years. Across groups, putative dates for first reproductive events of the season were identified for mating (August 19 to September 19), parturition (October 19 to November 19), and pup emergence (November 19 to December 19).

Mongooses were evicted from two groups in our study population (including one event previously recorded [82]). Both evictions occurred during October between the first group estrus and parturition of the season. Two other groups fused, also in October. Other inter-group agonistic encounters (tallied in parentheses) that we observed, occurred with greater frequency towards the end of the dry season: June (1); July (1); August (3); September (4); October (3—the evictions and fusion indicated above); November (2).

## Discussion

### Covariates explaining variation in fecal glucocorticoid metabolite levels

In our study, food limitation and reproduction best explained variability in fGCMs in banded mongoose at the population-level. In free-living groups, current food limitation (fecal organic matter), best explained variability in fGCMs. This food limitation effect was overwhelming in free-living mongooses, but only marginally better than a random intercept model in captive mongooses fed a regular supply of food. The effect was progressively larger for mongoose groups with less access to anthropogenic resources.

Free-living banded mongooses exposed to greater food limitation (groups with lower fecal organic matter and less access to anthropogenic food sources) exhibited higher fGCM levels. However, the fecal organic matter content of feces at the group level did not exhibit the overt seasonality exhibited in the fGCM levels. For our monitored mongooses that had access to anthropogenic food resources (most animals in the study), as natural food resources dwindled in the dry season, animals may have relied more heavily on anthropogenic food resources (thus maintaining relatively constant group-level organic matter intake year-round). The concentrated anthropogenic food resources may have resulted in increased agonism and may have caused food limitation in animals that lost agonistic encounters over concentrated food resources. This assertion is supported by evidence that home and core ranges shrink in the dry season around lodges and refuse sites where mongooses were observed feeding [39]. The only group to increase home and core ranges in the dry season in our study was a “park” group with no access to anthropogenic food resources [39]. We suspect that this group (for which we did not have a complete longitudinal fGCM profile) had to range further in the dry season to meet energetic requirements (lacking the supplementation from refuse sites) and we would predict that a strong seasonal pattern in fGCMs and possibly in fecal organic matter content would be present. Future research should focus on longitudinal fecal collection from such groups to further evaluate the influence of anthropogenic resources on fGCMs in transformed landscapes.

### Ecological context: timing, magnitude, and seasonality of stress responses

At the group level, peak glucocorticoid levels in free-living banded mongoose coincided approximately with the early breeding season but the amount and nature of anthropogenic food provisioning appeared to moderate the level and timing of fGCM peaks. Groups with dispersed and more abundant anthropogenic provisioning, had lower fGCM peaks, delayed until late dry season, coinciding with first parturition. Portions of these fGCM peaks may be explained by late pregnancy fGCM increases in subordinate females [50] or by fGCM increases associated with provisioning of pups [43]. However, these peaks were not repeated in subsequent parturition events later in the breeding season. More food-limited groups in the park with access to only one lodge had fGCM peaks that were 0.7-fold higher than those in groups with multiple lodges and refuse sites, and occurred earlier in the dry season, coinciding with first mating, group evictions, and group fissions. Once again, these peaks were not repeated later in the breeding season during periods of subsequent mating. Groups from the “park + lodge” and “urban + lodge” categories concentrated movements and foraging around lodge refuse sites [39] and these concentrated food sources increased aggression and agonistic encounters within social groups [83]. Free-living banded mongooses may therefore face a confluence of factors that increase dry season glucocorticoid levels—food limitation, agonistic encounters at concentrated food sources, aggressive evictions, estrus, competition for matings, parturition, subsequent predation of pups, and energetically-costly alloparental care. Access to more dispersed anthropogenic food sources may moderate and delay the combined effect of these factors. Increased wet season food availability may mitigate the effect of subsequent reproductive activity (including energetic losses associated with subordinate female pregnancies and pup provisioning) as the breeding season progresses. We acknowledge that our results, suggesting variability in the timing of seasonal fGCM peaks with differing levels of access to anthropogenic resources, are observational. Experimental studies are now required to determine the causal mechanisms behind this observed variability. Experimental manipulation in the field could involve closing access to lodge trash sites for some groups but not others for a before–after-control-intervention type of study. Similarly, food supplementation could be provided to some free-living groups. The amount, frequency, and dispersion of the supplemented food could also be varied to determine how single large “bonanza” anthropogenic resources might differ in their effect on mongoose fGCM levels, as compared to smaller, more frequent, and more dispersed supplemental resources that might not elicit agonistic encounters.

From these revealed patterns in fGCMs we can conclude that banded mongooses do exhibit seasonality in glucocorticoid production, as with the majority of mammal species [31]. The peak in this seasonal pattern for banded mongooses in northern Botswana does approximately coincide with early breeding season, a pattern that appears to diverge from other mammalian systems [31] and possibly banded mongooses in other study areas. If the preparative hypothesis [31] were true for banded mongooses (i.e. that glucocorticoid production should increase during periods when the likelihood alone and not necessarily the presence of stressors is increased, such as during a breeding season or resource-limited season) then we would have expected seasonality in glucocorticoids in the captive group even when food limitation and reproduction were controlled experimentally. This was not the case. Thus, only the energy mobilization hypothesis (glucocorticoid production should increase when energetic demands or deficits are greatest) and behavior hypothesis (glucocorticoid production should decrease during the breeding season) [31] are plausible explanations for seasonality in glucocorticoid production in banded mongooses. If anything, our study population and banded mongooses in Uganda [43, 50] exhibit increases in glucocorticoid production during the breeding season, not decreases. Given that the breeding season increases associated with provisioning pups and subordinate female pregnancies may ultimately be driven by energetic losses in the adult carers and subordinate females [43, 50], and our observation that breeding season peaks are unimodal i.e. not repeated at each breeding event in a breeding season, this would suggest more support for the energy mobilization hypothesis in explaining seasonality in glucocorticoid production in banded mongooses.

Free-living mongooses had chronic exposure to elevated glucocorticoids during some months. Our assessment used fGCMs which integrate the stress response over 24 h. Moreover, we collected feces from almost all animals within a group, thus integrating the response across animals. Our monthly fGCM estimates thus represent time periods that can be considered “chronic duration”. The magnitude of the stress response also suggests that the elevations at these times could be biologically meaningful: 19-, 12-, and 16-fold increases in peak months relative to a category’s (e.g. “park + lodge”) own nadir month or 38- and 66-fold increases in free-living groups’ peak months relative to a captive group’s nadir month all represent large effect sizes. More importantly, these among-group comparisons reflect only the median values for a given month. Free-living groups exhibited much higher variability in fGCMs within a month than the captive group, bordering on extreme variability in the case of the “park + lodge” category’s peak month. It seems plausible that at least some animals (exhibiting fGCMs in the upper quartiles) within a mongoose group entered homeostatic overload during the peak month.

### Putative epidemiological and fitness consequences of chronic fGCM elevations

Chronic exposure to elevated glucocorticoids may have epidemiological and hence fitness consequences for the animals that enter homeostatic overload. Glucocorticoids enhance, permit or suppress immune function [4] and may aid in redistribution of immune cells, specifically leukocytes and cytokines during immune responses [84–86]. However, chronic elevated glucocorticoid levels may cause allostatic load [6] and immune suppression—especially lowered skin immunity or cell-mediated immunity [84, 87]. Glucocorticoid and catecholamine effects on immune function are complex and involve suppression of cellular immunity and activation of humoral immunity. Cell-mediated immunity (suppressed by glucocorticoids) may confer resistance to intracellular pathogens such as mycobacteria, and *Mycobacterium tuberculosis* in particular [88].

Our banded mongoose population is infected with a novel *M. tuberculosis* complex pathogen, *M. mungi* [89]. *M. mungi* invades the mongoose host through breaks in the *planum nasale* and skin [90]. Outbreaks of *M. mungi* occur in multiple mongoose groups, infecting up to 17% of members in a social group (case fatality rate of 100%, [90]). The role of chronic glucocorticoid levels in the epidemiology of *M. mungi* is of particular interest because glucocorticoids are implicated in lowered skin immunity [84, 87], suppressed cellular immunity in general [2], and suppressed mycobacterial immunity specifically [91, 92]. During the late dry season, banded mongoose groups in our study area exhibit high fGCM concentrations, and for some individuals, extreme concentrations. Elevated fGCM concentrations at the group level appear to remain high for approximately 2 months, which might constitute “chronic” elevation. If so, the reactive scope (reactive homeostatic range) might be decreasing and the homeostatic overload threshold might be lowered in the short or long term. Hence, stressors that the mongooses could otherwise have mounted appropriate responses to, might now become pathological. Outbreaks of *M. mungi* occur predominantly in the dry season after an uncertain latent period, and it remains unclear how seasonal patterns of glucocorticoids might influence these disease dynamics. A future prospective study could follow the behavior and fGCM concentrations of uniquely-identified mongooses to better address this question.

### Anthropogenic provisioning as a putative ecological trap

Within the reactive scope model [5], we suspect that groups from the “urban + lodge” category show predictive homeostasis glucocorticoid responses to the combined effect of late dry season food limitation and first parturition (because median values in peak months differ only marginally from the captive group’s). Some animals in these groups may enter homeostatic overload at this time (because variability in this peak month is greater, with higher glucocorticoid levels than the captive group). More animals from the “park + lodge” category probably enter homeostatic overload during their peak month of glucocorticoid response. Anthropogenic provisioning at tourist lodges appears to help mitigate dry season food limitation in these groups. But we suspect that by focusing mongoose movements and foraging around a highly concentrated (but unpredictable) food resource, anthropogenic provisioning may present an ecological trap, inducing homeostatic overload, increasing injuries from agonistic encounters, and increasing potential for horizontal transmission of *M. mungi*.

### Study considerations and limitations

Our study is predominantly observational, and our results must be interpreted with that in mind. Although we were able to establish some causation in the association between reproductive activity and glucocorticoid production in our captive study animals, we were unwilling to limit the food of, or starve those animals to determine a causal link between food limitation and glucocorticoid production. Similarly, we could not prove without doubt a causal link between fecal organic matter content and food limitation. Perhaps future studies can circumvent this ethical quandary by finding large-scale natural experimental setups that can resolve this issue better.

Future studies should assess putative ecological covariates that we overlooked or use proxies that more accurately represent the covariates. Since our global model explained 55% of fGCM variability in free-living banded mongooses, other covariates (or more appropriate proxies) must account for unexplained variability. Some of the remaining variability might be explained by systematic biases associated with sex and age differences among the mongooses, and disease status—i.e. if certain categories of mongoose (e.g. older animals) either defecated in obscure sites that we missed, or if the sex ratio or age structure in a group changed over time. We did not account for such systematic biases in our sampling or models. We posit that grass height may be an additional important predation risk factor. Overall, our predictions about ecological covariates were generally upheld: the direction of association for each covariate matched our predictions, although the small effect sizes (for canopy cover) and large variability (soil macrofauna, breeding status, group size, canopy cover) of some covariates suggest that only three covariates (fecal organic matter, recent rainfall, access to anthropogenic resources) have strong predictive capability for explaining variability in fGCMs at the group level in banded mongooses in our study area.

One caveat is the potential effect of dietary differences (independent of food limitation) among banded mongoose groups on fecal glucocorticoid metabolism and excretion [93], and on glucocorticoid production [94]. We validated our fGCM assay using captive mongooses fed processed pet food [38]. Future studies should validate assays using mongooses fed ad libitum a variety of diets. However, we do not believe that dietary differences could explain the extreme variability in our data, either between captive and free-living mongooses or among free-living groups with access to different kinds of anthropogenic food resources. In red squirrels (*Tamiasciurus hudsonicus*), experimental changes in diet resulted in a 0.11-fold increase and a 0.14-fold decrease in fecal glucocorticoids [94]. If dietary differences had a similar effect in banded mongooses, the effect would be very small relative to the effect sizes that we observed.

## Conclusions

Free-living banded mongooses (and in particular, groups with access to anthropogenic food resources e.g. trash sites) in northern Botswana exhibit elevated glucocorticoid production over a period of weeks to months in the late dry season, likely pushing them into chronic homeostatic overload. Food limitation and access to anthropogenic food resources explain variability in glucocorticoid production at the population level for free-living banded mongoose groups (more fecal organic matter, and recent rainfall [affecting soil macrofauna availability] and hence lower food limitation, are associated with lower glucocorticoid levels; greater access to anthropogenic food resources is associated with lower glucocorticoid levels), while reproduction explains less and predation risk explains very little variability (glucocorticoid levels increase during reproductive periods; bigger groups and more canopy cover, and hence lower predation risk are associated with lower glucocorticoid levels). The energy mobilization hypothesis provides a plausible explanation for seasonality in glucocorticoid production in banded mongooses—glucocorticoid production increases when energetic losses or deficiencies occur. Free-living banded mongooses may face a confluence of factors that increase dry season glucocorticoid levels: food limitation, agonistic encounters at concentrated food sources, aggressive social group evictions, estrus, competition for matings, parturition, and subsequent predation of pups. Access to anthropogenic food sources appears to moderate the size and timing of these effects. As climate change will likely make this region hotter and drier, and as humans continue to expand their urban footprint in this region, the potential risk of homeostatic overload in banded mongooses will only increase. This homeostatic overload could have increasingly important fitness consequences. *M. mungi* disease outbreaks, an evolutionarily recent disease in this population, occur mainly in the dry season, providing an indication that important interactions between the environment, stress, host physiology, and disease dynamics might be occurring. Further studies are needed to understand these potential interactions in the epidemiology of *M. mungi* and to understand other possible consequences of homeostatic overload more generally in this species.

## Methods

### Study area and animals

Our study area was located in northern Botswana, a region characterized as a nutrient-poor, semi-arid savanna woodland. Annual mean rainfall was 552 mm (SD 148 mm, 1994–2006) falling predominantly in the rainy season (November to March, monthly mean > 50 mm) with little to no rainfall occurring in the dry season (May to September, monthly mean < 5 mm), and only sporadic rain during transition months (April, October; 5 mm $\le$ monthly mean $\le$ 50 mm).

Banded mongoose are small-bodied, diurnal, predominantly insectivorous herpestid carnivorans (< 2 kg). They are co-operative breeders [95] with limited social dominance [45] and low reproductive skew [96]. Females can conceive from the age of 290 days [97], have a gestation period of approximately 2 months [97] and estrus begins 10 days after parturition [97]. Pups emerge from dens to join adults on foraging forays at approximately 4 weeks of age [97]. Group evictions usually occur during group estrus events [44], possibly triggered by same-sex reproductive competition [46].

We studied 13 groups of free-living banded mongooses (group size: 5 to 64 individuals) and one group of captive mongooses (one female and three males) from October 2007 to November 2011. Our study groups occurred across a $\sim 120$ km$^{2}$ area that included the northeastern part of the Chobe National Park ($\sim 30$ km$^{2}$), northern Kasane Forest Reserve ($\sim 73$ km$^{2}$), and the towns of Kasane and Kazungula ($\sim 17$ km$^{2}$) at 25.163° E, 17.828° S [38]. These groups occurred along a gradient of association with humans (a synanthropic scale) [39], previously estimated using a singular value decomposition principal components analysis of tourist density (a proxy for food density in trash sites) and building density (a proxy for both anthropogenic food resources and denning opportunities) [39]. Here, we delineate social groups along that gradient in order of decreasing access to anthropogenic resources (and decreasing principal components score, PC1, for each social group [39]): “captive” (not applicable); “urban + lodge” (1.66, 1.22, 1.01, 0.83, 0.45); “park + lodge” (0.28, 0.00, − 0.92); “park” (− 2.18, − 2.36). These delineations are for illustrative purposes, and provide context for understanding the effect sizes that we report. They were not used in our modeling approach.

Captive animals, used in this study, were housed outdoors together at the CARACAL research facility in Kasane in an enclosure ($\sim 95\hbox {m}^{2}$), consisting of a perimeter wall of $\sim 1.5\hbox { m}$ height, enclosing habitat and substrate typical for the study area. Captive animals had been housed at the facility from 2 weeks of age and were 2 to 3 years old at the time of sampling. Captive animals were fed as a group with 820 g of canned wet pet food at 8 AM daily, a regimen established over several months at which the animals maintained their body condition. Individual consumption may have varied somewhat, but we did not detect dominance of the provisioned food by any particular animal. The captive animal diet was occasionally supplemented with natural prey items (coleopterans, spirostrepid millipedes, and bushveld rain frogs, *Breviceps adspersus*), and from foraging in their enclosure.

To develop a non-reproductive and non-food-limited control for wild banded mongoose evaluations, we suppressed reproduction in the captive group through the administration of an orally-delivered progestin contraceptive, megestrol acetate (Ovarid; Schering-Plough Corporation, Kenilworth, USA, September 2008 to May 2010) and maintained a regular food supply. During contraception, neither the males nor the female engaged in reproductive behavior, and no free-living mongooses visited this group. To then isolate the reproductive effect on fecal glucocorticoid production, we stopped contraception and allowed reproduction to occur. We observed estrous behavior and parturition in the captive female in addition to invasions by free-living mongooses, and pup depredation events during this period [38].

### Observations and sample collection

Fecal sample collection, transportation and storage methods have been previously described [38], as were our behavioral observation methods (e.g. movement, foraging, agonistic encounters, mate guarding, copulation) [98]. Briefly, we used telemetry homing to find and observe mongoose groups daily. We recorded movement, foraging, habitat, and social interaction data which were used in explanatory variables in our models. We collected $>6000$ fecal samples during the study, from which we selected a subset for further analysis. To make this final selection, we applied stratified sampling to provide optimal coverage across the study duration, spatially across the study area, and from among all available mongoose groups. For our final selection (Fig. 7) we analyzed 1542 fecal samples from our 13 free-ranging mongoose groups (138 sampling events, June 2008 to December 2010) and 202 fecal samples from our captive control group (68 sampling events, October 2008 to April 2011). From the free-living groups we collected a median of 59 samples (range: 3–584) over a median of six sampling events (range: 1–54) at a median interval (within a group) of 14 days (range: 1–282), from a median of 19 animals (range: 3–64). We collected feces within 4 h of defecation, the period that fGCM levels were determined to remain stable [38].

### Ecological covariates

Ecological covariates of fGCMs chosen *a priori* were either fecal bolus-specific (e.g. fecal organic matter) or related to potential stressors or resources encountered by the group (e.g. soil macrofauna and anthropogenic food availability, reproductive activity, and predation risk). In banded mongooses, fGCM excretion approximately matches gastrointestinal transit time (a minimum of 24 h) [38]. Because of our standardized morning fecal collection, we linked fecal samples to ecological covariates from 2 days prior (“covariate days”), when stressors would have elicited production of circulating glucocorticoids (based on gastrointestinal transit time [38], of at least 24 h). For each group’s covariate days, we plotted a “day range” using movement data [98]. We plotted a concave hull around multiple location estimates spanning multiple hours for a group on a covariate day. With sparse movement data or a single location estimate on a covariate day, we centered a circle on that location with area equivalent to the season-specific median daily minimum convex polygon for that group.

#### Food limitation—fecal organic matter

Assessing food limitation in free-living animals can raise important methodological challenges. Dry fecal matter contains organic and inorganic matter (or “total ash”), which we determined by ashing samples in a muffle oven [99]. Fecal inorganic matter could be from ingested food or substrate (e.g. soil). All steroid concentrations we report are expressed per mass of dry organic fecal matter [99] (i.e. we controlled for dilution of concentrations by ingested soil). By using percentage for fecal organic matter, we also removed bolus-size effects. In addition to controlling for steroid dilution, we modeled current food limitation using a bolus’ percentage organic matter. Higher organic matter content indicated higher food availability (and hence reduced food limitation).

Fecal organic matter increases with increasing organic matter or forage intake, increasing digestible organic matter intake, increasing nitrogen balance, and increasing nitrogen intake in cattle (*Bos taurus*) and goats (*Capra aegagrus hircus*) [56]. Fecal organic matter thus provides a good indication of nutritional status in ruminants if forage intake and forage digestibility are positively associated [56]. Further, several species are known to increase soil ingestion under food-limited conditions. Domestic sheep (*Ovis aries*) ingest more soil when forage availability [57] and feed supplementation decrease [58, 59], and when stocking rates increase [60].

Compared to herbivores, animals that eat soil macrofauna tend to consume even more soil [57], with larger animals such as myrmecophagus mammals ($> 1$ kg), ingesting soil indiscriminately with prey [100]. Soil ingestion increases as invertebrate prey availability declines for many insectivorous species. In seasons when their preferred termite prey, *Trinervitermes* sp., are scarce, aardwolves (*Proteles cristata*) exhibit more sand in their feces [61]. Captive tamanduas (*Tamandua tetradactyla*) ingest more substrate when they experience behavioral or dietary deficits [62]. Three-banded armadillos (*Tolypeutes tricinctus*) had no soil in their early wet season feces, when termite alates erupted, but ingested large quantities of soil during the dry season (as seen when comparing wet and dry season stomach contents *post mortem*) [63]. In giant anteaters (*Myrmecophaga tridactyla*) fecal nutrition markers (such as gross energy, organic matter, ether extract, crude fiber, crude protein, neutral detergent fiber, and nitrogen-free extract) all decreased as fecal total ash and acid-insoluble ash increased [64].

Soil ingestion and insectivory both have ecologically-relevant metabolic consequences and could affect glucocorticoid production. Insectivory, and myrmecophagy in particular, involves prey items of low nutritional value [101] that may be seasonally unavailable [102, 103] and result in much soil ingestion [61, 104]. Energy expenditure may be limiting in insectivorous animals, resulting in lower basal metabolic rates than predicted by body size [105, 106]. This lowered basal metabolic rate could be related to seasonal food limitation [106, 107], foraging habits [106, 107], low energy density of prey, and ingested soil, which further lowers energy density [100].

Food limitation and soil ingestion can be difficult to measure in free-living animals, as we describe elsewhere [39]. Fecal acid-insoluble ash may provide a reliable marker for soil ingestion [57], as it does for 28 wildlife species assessed [108]. To determine whether total ash may provide a marker equivalent to acid-insoluble ash, we estimated both in 30 of our samples, and found a strong correlation between the two measures (Pearson’s *r* = 0.94). Dietary ash may skew estimates of soil ingestion from fecal ash. A few invertebrates (earthworms, geophagous termite workers, and termite soldiers) have high ash content [101], but invertebrates generally have high digestibility (78%) and low total ash content (5%) [109]. Soil may form 20% to 30% of earthworm dry weight [57], but earthworms are absent from dry savannas, none were found while sampling invertebrates in our study area [66], and none were recorded in Ugandan banded mongoose diets [65]. For typical banded mongoose prey items, ash content is low e.g. termite alates (7%) [110]. Captive mongooses were fed food with total ash content of 7 to 9%. Mineral soil generally has > 90% ash content [108]. Fecal ash (or conversely, fecal organic matter) may thus provide a reasonable marker for soil ingestion and food limitation in our study [39].

#### Food limitation—soil macrofauna density

At coarse spatio-temporal scales (e.g. the scale of seasons over large habitat patches), banded mongoose food limitation can be affected by soil macrofauna availability and anthropogenic food. We recorded and digitized habitat zones for the site from 3600 direct behavioral and clinical observations of mongoose groups (after telemetry homing). We used previously-described habitat classifications associated with season- and habitat-specific soil macrofauna density (m$^{-2}$) [66] estimates for our study area. For the day range used by a mongoose group on a given day, we multiplied habitat areas within the day range by corresponding seasonal habitat-specific macrofauna densities [66] as an estimate of macrofauna density across the day range.

#### Food limitation—rainfall

Rainfall could affect soil macrofauna availability over short spatio-temporal scales (e.g. availability over the scale of a few days in response to local movement of macrofauna). Rainfall causes soil macrofauna to migrate upwards in soil and increases their wet season availability in our study area [66]. Further, two important mongoose foods in our study area respond to rainfall, increasing their availability: termite alates erupt for ‘nuptial flights’ at the first substantial rainfall of the season [68] and spirostreptid millipedes forage on the ground surface after rain [67]. Four of ten feces analyzed after an alate eruption in Uganda “consisted almost entirely of termite reproductives” [65], and millipedes were found in 96% of banded mongoose feces analyzed for diet, making up 76% of the volume of feces, except during the dry season [65]. To model the effect of rainfall and soil macrofauna availability on mongoose fGCM concentrations, we summed rainfall measured at a centrally-located meteorological station for 7 days before each “covariate day”, a period chosen based on data for millipede activity in southern Botswana [67]: the median number of consecutive days after the rain with millipede activity was 2 (range: 0–8). We used these summed rainfall values as a covariate for modeling mongoose fGCM concentrations.

#### Food limitation—anthropogenic food resources

In Uganda, refuse-feeding groups had smaller core ranges than non-refuse-feeding groups [55], and adults from refuse-feeding groups were heavier and exhibited better physical condition [54]. At our study site, banded mongooses foraged in refuse in 110 of 850 (13%) foraging observations and drank from anthropogenic water sources in 78% of all observations of mongoose drinking behavior [39]. Synanthropic groups (those living in association with humans) had more concentrated home and core ranges, which were centered on tourist lodges and trash sites [39]. There was also a high certainty (20:1 odds) that the level of a social group’s association with humans was positively associated the group’s median fecal organic matter content [39]. Thus, we modeled access to concentrated anthropogenic food resources as a binary factor covariate in our models of mongoose fGCM concentrations: “yes” if day ranges overlapped tourist lodges or substantial refuse sites, otherwise “no”.

#### Reproductive activity

We delineated putative dates for estrus and mating for each group from behavioral observations of mate guarding and copulations. When we lacked observations of mating behavior, we used pup emergence to estimate parturition (4 weeks prior) and conception dates (3 months prior) based on well-established time to pup emergence and length of gestation for banded mongooses in Uganda [97]. We assigned a binary variable for breeding status (1, breeding; 0, non-breeding) for fecal samples based on the putative or observed dates for mating and parturition, i.e. we assigned a 1 if we obtained fecal samples within a few days of a reproductive event. These “reproduction” fecal samples represented 15 distinct reproductive events for nine mongoose groups from 2008 to 2010, and included 224 fecal samples. The median delay between the reproductive event and the fecal sample collection as 2 d (inter-quartile range: 3 – 1.5 d), which was appropriate given the minimum gastrointestinal transit time of 24 h [38].

#### Predation risk—canopy cover

Published depredations of banded mongooses are predominantly due to avian predators such as martial eagles (*Polemaetus bellicosus*—Serengeti) [77] and marabou storks (*Leptoptilos crumeniferus*, 50% of known mortalities—Uganda) [54]. In Uganda, banded mongooses also mob fish eagles (*Haliaeetus vocifer*) [77]. Reptiles (12.5%), carnivores (12.5%), humans (12.5%), and warthogs (*Phacochoerus africanus*, 12.5%) were other causes of known depredations of banded mongooses in Uganda [54]. In our study area, we recorded 55 adult mortalities of known cause in 2008 and 2009 [39]. Of these mortalities, most were disease- or urban-associated: *M. mungi* infection (45%), humans (36%, including roadkill), and domestic dogs (9.1%) [39]. Natural depredations accounted for the remaining mortalities: raptors (7.3%) and carnivores (1.8%) [39].

We assumed that canopy cover conferred protection from aerial predators (the primary natural predator for adult banded mongoose in our system). Thus, with greater canopy cover, perceived predation risk by banded mongooses should be lower, and hence, fGCM concentrations should be lower. Banded mongooses commonly occur in riparian zones in southern Africa, presumably due to the physiognomy of the vegetation [111], and possibly for predation refuge. To evaluate this potential influence, we digitized canopies of 62,000 trees and bushes from satellite imagery (Google Earth, Google Inc., Mountain View, CA, USA) and estimated percentage canopy cover for each day range, and used that canopy cover as a covariate in our models of fGCM concentrations.

#### Predation risk—group size

Larger group sizes should also lower per capita vigilance [74, 75], which has been shown experimentally for banded mongooses [76]. Group size could thus be used as an additional proxy for predation risk. We estimated group size (adults) by counting adults during behavioral observations on multiple days each month (median of 4 counts per month, range: 1–43). Mongooses forage in groups but some animals may guard pups or forage separately. Thus, we used the maximum number of adults counted consistently each month as a covariate in our models of mongoose fGCM concentrations.

### Steroid extraction and analysis

We conducted steroid extraction and analysis as previously described [38]. Briefly, we lyophilized, pulverized and sifted fecal samples to remove fibrous material [19], and then extracted $\sim 0.05$ g of fecal powder with 80% ethanol in water (1.5 ml) [19]. We measured extracts for immunoreactive fGCMs using an enzyme immunoassay (EIA) on microtiter plates [112], detecting 11,17-dioxoandrostanes (11,17-DOA). Intra-assay coefficient of variation (CV) for this test was 2.8–4.0% and inter-assay CV was 12.1–16.8%. Assay sensitivity at 90% binding was 3 pg/well.

### Model building and selection

To explore population-level ecological covariates, we used *a priori* mixed-effects models to evaluate fGCM concentration in free-living mongooses. We include our dataset as a Additional file 1. For balanced-design model averaging, we used all global model subsets (all were plausible) and omitted interaction terms. Our global model had seven fixed effects: proportion of fecal organic matter in a bolus (“org”); access to concentrated anthropogenic food sources (“anth”); rainfall amount over the previous 7 days (“rain”); percentage canopy cover (“cc”); group size (“size”); group breeding status (“breed”); density of soil macrofauna (“macro”). Individual identity of feces was unknown and hence our inferences were made at the group and population levels. We modeled sampling event (1|event) as a random effect, controlling for repeated measures on the same day for a given mongoose group. We also modeled group identity (1|group) as a random effect, with the 13 groups assumed to represent a random sample of groups from the overall population, controlling for multiple sampling events for a given mongoose group.

To test for group-level effects of anthropogenic food provisioning and the validity of using fecal organic matter as proxy for food limitation, we developed *a priori* models for fGCM concentration in a single free-living group and the captive group. The particular free-living group was chosen because its home range was in the immediate vicinity of the captive enclosure—the two groups thus had similar habitat and rainfall. Our sample collection schedule for this group also matched the sample collection schedule for the captive group. The median sampling interval for the captive group was 8.5 days (inter-quartile range: 4–15.5 days). The median sampling interval for the free-living group was 10 days (inter-quartile range: 4–14.5 days). Only fecal organic matter (org., fixed effect) and sampling event (1|event, random effect) varied in the captive group. Although rainfall varied for the captive group, we deemed this unimportant because the captive mongooses derived little of their diet from foraging in the enclosure. Thus, we used two candidate models for the two groups: a mixed-effects model (fecal organic matter and sampling event), and a random-effects model (sampling event). We expected fecal organic matter to describe fGCMs in the free-living but not the captive group.

We modeled fGCM concentrations (natural log transformed) as the response variable in linear mixed models fitted with the ‘identity’ link function using *lmer* in Package ‘lme4’ in R [113]. We standardized numeric variables to $\bar{x} = 0, \sigma = 0.5$ and binary variables to $\bar{x} = 0$ with a difference of 1 between categories [114], using Package ‘arm’. We assessed multicolinearity with variance inflation factors (VIFs) [115], using *a priori* rule-of-thumb guidelines of VIF $=4$ (moderate multicolinearity) and VIF $=10$ (extreme multicolinearity) for interpretation, but acknowledging the context for the analysis [116]. We evaluated candidate models using Akaike’s Information Criterion [117] with small sample size correction (AIC$_c$) [118]. We used multimodel inference and model averaging [119] with Akaike weights ($w_i$) for all candidate models. We used 85% confidence intervals [80, 118] to assess goodness of fit of parameter estimates and $\Omega _0^2$ to assess variation explained by the global model [120]. Model selection using AIC has a slightly higher chance (1 in 6) of selecting spurious or uninformative variables, than does hypothesis testing with $\alpha = 0.05$ [80], hence our use of 85% confidence intervals, which more closely matched the potential error rate under AIC. However, we tried to prevent overfitting and exclude uninformative variables through a combination of measures: selecting a reasonable set of candidate variables *a priori*, performing model averaging, and interpreting Akaike weights and variable importance.

To assess the association between fecal organic matter and fGCM concentration (natural log transformed), we used Bayesian linear regressions, conducted in Package ‘rstan’. To assess seasonal differences in fecal organic matter and fGCM concentrations (again, natural log transformed), we used Bayesian estimation of wet season minus dry season values (equivalent to a classical *t* test), conducted in Package ‘BEST’. For all Bayesian analyses, we used weakly informative priors, we used sufficient burn-in and checked traceplots for model convergence. We report the mean of the posterior distribution and the 95% highest posterior density interval (HPDI).

Where applicable, we used quartile plots [81] to display data with medians depicted as points, values within 1.5*(inter-quartile range) of first and third quartiles depicted with lines (bounded by lower and upper extremes), and inter-quartile range left clear or depicted with lines of contrasting color (bounded by first and third quartiles). We depict *x*-fold changes in fGCM concentrations to illustrate the magnitude of effects, whereby *x*-fold change = (larger value − smaller value)/smaller value.

## Supplementary information

**Additional file 1.** Raw data for fecal glucocorticoid metabolites and candidate variables from free-ranging banded mongooses in northern Botswana.

## Data Availability

The dataset supporting the conclusions of this article is included within the article and its Additional file 1.

## Abbreviations

11,17-DOA: 11,17-Dioxoandrostanes
AICc: Akaike’s information criterion with small sample size correction
anth: Access to concentrated anthropogenic food sources
cc: Percent canopy cover
CV: Coefficient of variation
EIA: Enzyme immunoassay
fGCM: Fecal glucocorticoid metabolite
GC: Glucocorticoid
IFN-$\gamma$: Interferon-gamma
IL-12: Interleukin-12
macro: Soil macrofauna density
org: Percent fecal organic matter
VIF: Variance inflation factor
